# Label-Free Screening of SARS-CoV-2 NSP14 Exonuclease Activity Using SAMDI Mass Spectrometry

**DOI:** 10.1177/24725552211008854

**Published:** 2021-04-17

**Authors:** Michael D. Scholle, Cheng Liu, Jerome Deval, Zachary A. Gurard-Levin

**Affiliations:** 1 SAMDI Tech, Inc., Chicago, IL, USA; 2 Aligos Therapeutics, Inc., South San Francisco, CA, USA

**Keywords:** COVID-19, coronavirus, NSP14, nuclease, mass spectrometry, label-free

## Abstract

Severe acute respiratory syndrome coronavirus 2 (SARS-CoV-2) is the virus responsible for the global COVID-19 pandemic. Nonstructural protein 14 (NSP14), which features exonuclease (ExoN) and guanine N7 methyltransferase activity, is a critical player in SARS-CoV-2 replication and fidelity and represents an attractive antiviral target. Initiating drug discovery efforts for nucleases such as NSP14 remains a challenge due to a lack of suitable high-throughput assay methodologies. This report describes the combination of self-assembled monolayers and matrix-assisted laser desorption ionization mass spectrometry to enable the first label-free and high-throughput assay for NSP14 ExoN activity. The assay was used to measure NSP14 activity and gain insight into substrate specificity and the reaction mechanism. Next, the assay was optimized for kinetically balanced conditions and miniaturized, while achieving a robust assay (Z factor > 0.8) and a significant assay window (signal-to-background ratio > 200). Screening 10,240 small molecules from a diverse library revealed candidate inhibitors, which were counterscreened for NSP14 selectivity and RNA intercalation. The assay methodology described here will enable, for the first time, a label-free and high-throughput assay for NSP14 ExoN activity to accelerate drug discovery efforts and, due to the assay flexibility, can be more broadly applicable for measuring other enzyme activities from other viruses or implicated in various pathologies.

## Introduction

Severe acute respiratory syndrome coronavirus 2 (SARS-CoV-2) is the cause of the continuing global pandemic that began in 2019, commonly termed COVID-19. SARS-CoV-2 is an enveloped, positive-sense, single-stranded RNA (ssRNA) virus that belongs to the β-coronavirus genus of the Coronaviridae family, which also includes SARS and MERS β-coronaviruses.^1^ Viral RNA synthesis and subsequent processing are regulated by the nonstructural proteins (NSPs) 7 to 16 after cleavage by the viral 3CL protease (3CLpro).^2^ Significant progress has been made in vaccine development, while small-molecule therapeutics as treatment or prophylactics remain a critical unmet need. NSP14 plays a critical role in SARS-CoV-2 replication^3^ and has emerged as an attractive therapeutic target for SARS-CoV-2. NSP14 is a bifunctional enzyme featuring 3′-5′ exonuclease (ExoN) as well as guanine methyltransferase (N7-MTase) activities. NSP14 ExoN activity is implicated in a proofreading mechanism on dsRNA to remove nonengineered mutations from dsRNA to ensure sequence integrity.^4–6^ The ExoN activity is enhanced when NSP14 is in a complex with the NSP10 protein, and the complex operates to repair misincorporations during RNA synthesis.^7,8^ Remarkably, NSP14 efficiently excises the nucleoside analog ribavirin,^9^ an antiviral drug, consistent with the finding that the drug is less effective in CoVs.^10,11^ Genetic studies further support the therapeutic potential of NSP14. Loss-of-function ExoN mutants exhibit a 20-fold increase in replication mutations,^12,13^ rendering the virus more sensitive to drug-induced lethal mutagenesis.^14^ NSP14 ExoN activity also digests double-stranded (dsRNA)—an intermediate of viral replication that often triggers an immune response—suggesting that NSP14 may shield the RNA from recognition by innate immune sensors.^15^ These data point toward small-molecule inhibitors of NSP14 as a promising antiviral therapeutic strategy for SARS-CoV-2 and potentially other viruses. To date, reported small-molecule inhibitors against NSP14 lack selectivity and do not offer opportunities for structure–activity relationship optimization and medicinal chemistry efforts.^16^

Large-scale drug discovery efforts require robust, high-throughput, and high-quality assays to be developed. Nuclease assays are well described, often through the incorporation of radiolabeled phosphate and processing through polyacrylamide gel electrophoresis methodologies.^6–8,17^ These approaches are cumbersome to prepare the substrate, requiring special handling, safety precautions, and dedicated waste removal, while also lacking the high-throughput capability. Alternatively, fluorescent assays have emerged that use intercalation dyes that are removed upon nuclease activity, leading to a loss of signal.^18^ While the assays offer a high-throughput readout and convenient reagents, these assays are prone to high rates of false-positive and/or false-negative results due to optical interference of library compounds.

This study reports the development of the first label-free, high-throughput assay for measuring NSP14 ExoN activity. The approach combines self-assembled monolayers of alkanethiolates on gold with matrix-assisted laser desorption ionization time-of-flight (MALDI-TOF) mass spectrometry (MS). This methodology, termed SAMDI-MS (self-assembled monolayer desorption ionization MS), overcomes the limitations of traditional MALDI-TOF-MS and other MS approaches. The specific immobilization of the analyte(s) of interest to the monolayer, while all other components are washed away, enables the analysis of virtually any enzyme activity that results in a mass shift. The ability to detect multiple reaction products can shed light on enzyme mechanisms. Moreover, the platform is amenable to any buffer system, including detergents, high salts, carrier proteins, organic additives, and even cell lysates.^19–21^ SAMDI-MS has been reported to measure biochemical activities on peptides,^22–24^ DNA,^25^ and small molecules,^26–29^ and has recently been validated on RNA substrates.^30^ A major benefit of SAMDI-MS for RNA substrates is the ability to monitor the purity and integrity of RNA in every reaction well, where RNases present in most labs and/or enzyme preps can lead to RNA degradation and false positives/negatives in label-based approaches such as Forster resonance energy transfer. The NSP14 ExoN assay was biochemically characterized to ensure conditions were in a kinetically relevant range, including assessing linear enzyme activity and determining substrate *K*_M_ values. The SAMDI-MS assay was used to screen 10,240 compounds, and hits were counterscreened and evaluated against RNaseT1 and for RNA intercalation. The advantages of the SAMDI-MS assay over traditional assays and implications for improved drug discovery efforts are discussed.

## Materials and Methods

### Proteins and Compounds

Codon-optimized SARS-CoV-2 NSP10 and NSP14 genes were synthesized and cloned into pET-Duet-1 vector (Millipore Sigma, Burlington, MA). NSP10 was fused to an N-terminal Strep-tag II, and NSP14 was fused to an N-terminal 8xHis tag similar to that described for SARS-Cov-1 NSP14/NSP10.^7^ An expression plasmid for ExoN-activity-dead mutant (D273A) was generated by site-directed mutagenesis. The wild-type (WT) SARS-CoV-2 NSP14/NSP10 and mutant (D273A) were expressed in *Escherichia coli* BL21 (DE3). The expression was induced by 0.5 mM IPTG at 16 °C for 20 h. Cells were harvested and cell pellets were disrupted in lysis buffer (50 mM HEPES, pH 7.5, 300 mM NaCl, 20 mM imidazole, 4 mM MgCl_2_, 0.5 mM TCEP, 5% glycerol) supplemented with protease inhibitor cocktail (Roche, Basel, Switzerland). The clarified lysates were purified by an Ni-NTA column. The elution pools from the Ni-NTA column (Qiagen, Hilden, Germany) were dialyzed against lysis buffer and purified on a high-resolution Ni column (HiTrap IMAC HP, GE, Marlborough, MA). The protein complexes were further purified on a size exclusion column (Superdex 200 [16/60] column, GE) equilibrated with SEC buffer (20 mM HEPES, pH 7.5, 200 mM NaCl, 2 mM MgCl_2_, 0.5 mM TCEP, 5% glycerol). RNA substrates and the internal standard were purchased from Integrated DNA Technologies (Coralville, IA) and purified to >95% by high-performance liquid chromatography (HPLC). RNaseT1 was purchased from Thermo Fisher Scientific (Waltham, MA). All other chemicals were purchased from Sigma Aldrich (St. Louis, MO).

### NSP14/NSP10 ExoN MS Assay

NSP14 assays were performed in 6 µL volumes in 384-well low-volume polypropylene microtiter plates (Greiner Bio-One, Kremsmünster, Austria; cat. 784201) at ambient temperature. The optimized assay buffer was 40 mM Tris-HCl, pH 7.5, 0.01% Tween-20, 0.01% bovine skin gelatin (BSG), 1 mM MgCl_2_, 5 mM DTT. For compound screening, NSP14/NSP10 (final concentration, 100 pM) was added using a Multidrop Combi (Thermo Fisher Scientific) and preincubated for 30 min with small molecules to allow for slow on rates. Reactions were initiated by the addition of the RNA duplex substrate (final concentration, 100 nM) and incubated for 60 min. Reactions were quenched by the addition of 100 mM EDTA (final). The reaction included an internal standard (100 nM final). For SAMDI-MS analysis, 2 µL of each reaction mixture was transferred using a 384-channel automated liquid handler for SAMDI biochip arrays functionalized with a neutravidin-presenting self-assembled monolayer, as previously reported.^30^ The SAMDI arrays were incubated for 60 mins in a humidified chamber to allow the specific immobilization of the biotinylated RNA substrate and product along with the biotinylated internal standard. The samples were purified by washing the SAMDI arrays with deionized ultra-filtered water (50 µL/spot) and dried with compressed air. A matrix comprising 2-hydroxy-5-methyoxybenzoic acid in acetonitrile (30 mg/mL) and ascorbic acid in aqueous ammonium citrate (500 mM) was applied by dispensing 350 nL on each spot in the array. SAMDI-MS was performed using the reflector-negative mode on an AB Sciex (TOF/TOF) 5800 MALDI mass spectrometer (Framingham, MA) with a laser intensity of 4000 using 324 shots/spectrum in a random raster sampling (18 shots/subspectrum with 18-subspectrum pass acceptance), 400 Hz laser frequency, bin size of 1 ns, and detector voltage multiplier of 0.56. A mass window of *m*/*z* 1600 to *m*/*z* 5000 was used, and a mass threshold of 0.5 Da applied. Areas under the curve (AUCs) were generated using the OEM Applied Biosystems Series Explorer software, and the amount of product generated was calculated using the ratio of product AUCs divided by the sum of the AUC of the internal standard and products. Assay robustness was determined by the Z factor,^31^ calculated using the following equation: Z factor = 1 – 3(σ_+_ + σ_–_)/(µ_+_ – µ_–_), where σ is the standard deviation and µ is the average conversion of positive (+) and negative (–) controls.

### Data Analysis

GraphPad Prism (San Diego, CA) was used to calculate enzyme kinetics and parameters such as *K*_M_ and *k*_cat_. Michaelis–Menten fits of steady-state enzyme velocities were applied in calculations. IC_50_ values and Hill slopes were generated using a four-parameter fit.

### High-Throughput Screening

The 10,240 compounds included in this screen (SAMDI Tech collection) are derived from a diverse compound library synthesized in 2020 and dissolved in DMSO, with each compound achieving an average purity >95%. PAINS (pan-assay interference compounds) have been eliminated from the library, and the compounds have an average molecular weight of 322 Da and LogP of 1.799. Compounds were screened in pools of 8 (final concentration, 12.5 µM for each compound) by stamping 60 nL of 1.25 mM compounds into 384-well plates using a Mosquito HTS Automated Liquid Handler (SPT Labtech, UK). The assay was performed as described above. A selection of compounds was reassessed by assaying each compound individually in the NSP14/10 assay, along with RNaseT1 and an RNA intercalation assay. The 100% inhibition controls using 100 mM EDTA (final) were placed in 32 wells, and 0% inhibition controls of 60 nL DMSO were added to another 32 wells. Assay quality was monitored using the Z factor and signal-to-background (S/B) ratio for each 384-well plate.

### Counterscreen Assays

Validated hits were further tested against RNaseT1 and in an RNA intercalation assay to rule out nonspecific RNA nuclease inhibition and RNA intercalation, respectively. RNA intercalation was assessed using a Thiazole Orange assay in 20 µL volumes in 384-well microtiter plates (Greiner Bio-One; cat. 781900) at ambient temperature. The optimized buffer was 40 mM Tris, pH 8.0, 1 mM MgCl_2_, 0.01% BSG, 0.01% Tween-20, 1 mM DTT. The dsRNA substrate used in the high-throughput screening (HTS) was incubated with compounds over a range of concentrations (25 µM to 0.3 µM) for 30 min, and reactions were initiated by the addition of 1 µM (final) Thiazole Orange and incubated for 30 min. RNA intercalation was assessed on a Pherastar FS plate reader (BMG Labtech) using an excitation 485 nm/emission 520 nm filter and results were compared with a mitoxantrone control with a robustness (Z′ factor) of 0.800. RNaseT1 assays were run in 20 µL volumes in 384-well low-volume polypropylene microtiter plates (Greiner Bio-One; cat. 784201) at ambient temperature. The optimized assay buffer was 20 mM HEPES, pH 7.5, 20 mM KCl, 0.01% Tween-20, 0.01% BSG, 1 mM DTT. RNaseT1 (final concentration, 0.002 U/µL) was added using a Multidrop Combi (Thermo Fisher Scientific) and preincubated for 30 min with small molecules to allow for slow on rates. Reactions were initiated by the addition of the ssRNA substrate (final concentration, 100 nM) and incubated for 5 min. Reactions were quenched by the addition of 0.5% formic acid (final). The reaction included an internal standard (100 nM final). For SAMDI-MS analysis, 2 µL of each reaction mixture was transferred using a 384-channel automated liquid handler to SAMDI biochip arrays functionalized with a neutravidin-presenting self-assembled monolayer, as previously reported. The robustness of this counterscreen assay (Z′ factor) was 0.671.

## Results

### Development of an MS Assay of NSP14/NSP10 ExoN Activity

**Figure 1.**
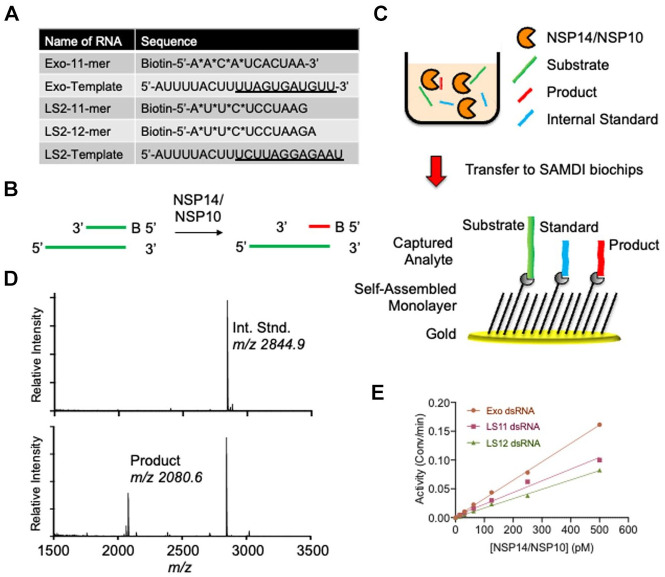
SAMDI-MS assay of NSP14 ExoN activity. (**A**) RNA substrates utilized in this study. Asterisk represents a PO bond. (**B**) Scheme of the NSP14/NSP10 ExoN assay on dsRNA substrates, where B represents the biotin moiety. (**C**) Scheme of SAMDI-MS assay where quenched homogenous reactions are transferred to neutravidin-presenting self-assembled monolayers to specifically immobilize the biotinylated substrate, products, and internal standard. (**D**) Representative SAMDI-MS spectrum showing the internal standard (*m*/*z* 2844.9) and major nuclease product using the Exo control substrate (*m*/*z* 2080.6). (**E**) NSP14/NSP10 exhibits ExoN activity on three substrates measured by SAMDI-MS. All measurements are from triplicate data.

To develop a SAMDI-MS enzymatic assay for NSP14/NSP10 ExoN activity, we designed 5′-overhang dsRNA substrates based on sequences previously described^9^ (**Fig. 1A**). As NSP14 ExoN activity acts in a 3′-5′ direction, the substrate strand incorporates a 5′-biotin group that allows for the specific immobilization onto neutravidin-presenting self-assembled monolayers for SAMDI-MS analysis. Upon ExoN activity, the enzyme will digest in a 3′-5′ direction, such that the substrate and all products will be immobilized through the biotin moiety (**Fig. 1B**). The reaction is set up by adding the dsRNA substrate to the enzyme in a 384-well plate. At the desired time point, the reaction is quenched by the addition of 100 mM EDTA (final). Automated liquid handlers then transfer 2 µL of the reactions to neutravidin presenting SAMDI biochip arrays to specifically immobilize the substrate, product, and internal standard (**Fig. 1C**). To assess product yield in an MS assay, it is important to consider ionization efficiency of the substrate and products. To facilitate quantitative analysis, the substrates feature nonhydrolyzable phosphorothioate (PO) bonds at the six terminal 5′-nucleotides, enabling the generation of a six-nucleotide product. Second, a six-nucleotide DNA internal standard was designed that mimics the anticipated RNA product in ionization potential and size, although with a distinct mass. This enables a quantitative analysis of product yield by SAMDI-MS. Indeed, upon ExoN activity, the spectra revealed the internal standard peak at *m*/*z* 2844.9 and a product at *m*/*z* 2080.6, corresponding to the six-nucleotide anticipated oligo (**Fig. 1D**). The amount of product observed is calculated by dividing the AUC of the product by the sum of the AUCs of the internal standard and product. Importantly, NSP14/NSP10 exhibits similar ExoN activity on multiple dsRNA substrates (**Fig. 1E, Suppl. Fig. S1A,C,E**). Also, NSP14/NSP10 failed to digest ssRNA substrates (**Suppl. Fig. S1B,D,F**), supporting the mechanism that NSP14/NSP10 acts specifically on dsRNA substrates.^6^ Taken together, the data suggest that the SAMDI-MS technology is well suited for measuring and reporting on NSP14/NSP10 ExoN activity.

### NSP14/NSP10 WT and Mutant Substrate Specificity

**Figure 2.**
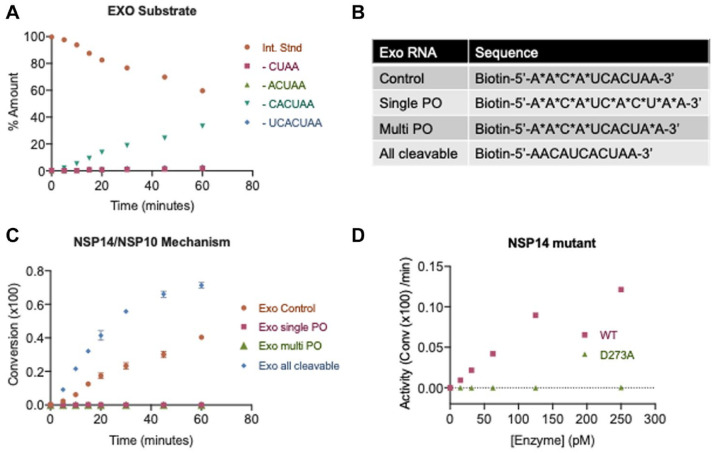
NSP14/NSP10 substrate specificity and reaction mechanism. (**A**) NSP14/NSP10 activity on the Exo control substrate results primarily in one product. (**B**) Sequences of distinct RNA substrates that pair with the Exo template. Asterisks represent PO bonds. (**C**) NSP14/NSP10 activity measured by SAMDI-MS on RNA substrates in **B**. (**D**) The NSP14 D273A mutant is not active against the Exo control substrate. All measurements are from triplicate data.

One of the benefits of using SAMDI-MS to measure ExoN activity is the ability to distinguish multiple products. Upon treatment of the Exo control substrate with NSP14/NSP10, the major product corresponds to the loss of CACUAA (**Fig. 2A**), consistent with the placement of the nonhydrolyzable linkers. Interestingly, upon exchanging the Exo control substrate for a substrate lacking the nonhydrolyzable PO bonds (herein “all cleavable”), multiple products are observed in the SAMDI-MS spectra (**Suppl. Fig. S2A,B**). To ensure that the observed activity is due to NSP14/NSP10 and not any other nuclease contaminant, the enzyme was tested against a series of dsRNA substrates that featured nonhydrolyzable PO bonds at different sites within the sequence (**Fig. 2B**). Consistent with earlier results, NSP14/NSP10 is active on the Exo control and all cleavable substrates (**Fig. 2C**). Notably, an Exo sequence featuring all PO bonds with the exception of a single internal nucleotide is not active against NSP14/NSP10, supporting that the activity is not due to contaminating endonuclease activity (**Fig. 2C**). Moreover, an Exo sequence featuring a PO bond on the first 3′ NT is also not active against NSP14/NSP10, confirming that the enzyme acts in a 3′-5′ direction and that the first nucleotide must be digested before proceeding with subsequent activities (**Fig. 2C**). Finally, the activity of WT NSP14/NSP10 was compared with a catalytically inactive mutant NSP14, D273A.^3^ SAMDI-MS fails to observe any nuclease activity with the catalytic mutant (**Fig. 2D**, **Suppl. Fig. S2D,E**), further supporting that ExoN activity is specific to the active form of the enzyme and not to contaminating nucleases.

### Assay Development and Kinetic Parameters

**Figure 3.**
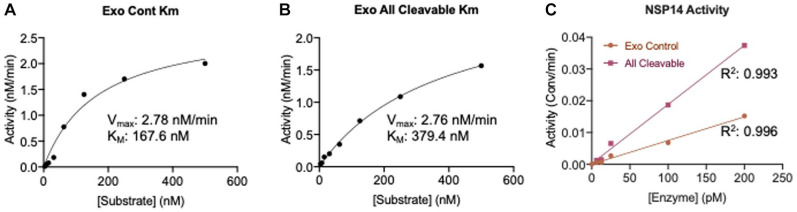
Kinetic parameters determined by SAMDI-MS. The *K*_M_ values of the (**A**) Exo control substrate and (**B**) Exo all cleavable substrate are determined by SAMDI-MS. (**C**) NSP14/NSP10 activity is linear to 200 pM using 100 nM of each substrate. All measurements are from triplicate data.

To develop an assay suitable for identifying potential small-molecule inhibitors, it is important to optimize the assay conditions and determine the kinetic parameters, including the substrate *K*_M_. The *K*_M_ was determined by testing NSP14/NSP10 against the Exo control and Exo all cleavable substrates over a concentration range of 500 nM to 7.5 nM (**Suppl. Fig. S3A,B**). The initial velocities (*V*_0_) were calculated using the linear portion of the reaction and fit to a Michaelis–Menten curve. The data suggest *K*_M_ values of 167.6 nM (95% CI, 86.51–356.4 nM) and 379.4 nM (95% CI, 297.5–495.3 nM) for the Exo control and all cleavable substrates, respectively (**Fig. 3A,B**). These data represent the first insight into the kinetic parameters of NSP14 ExoN activity. A final substrate concentration of 100 nM (below the *K*_M_) was chosen to enable the identification of small-molecule inhibitors that act through substrate competition. Incubating NSP14/NSP10 with 100 nM of each substrate highlights that the enzyme is linear to 200 pM (**Fig. 3C**, **Suppl. Fig. S3C,D**). Based on these data, 100 pM NSP14/NSP10 was selected for subsequent analysis.

### Assay Performance

**Figure 4.**
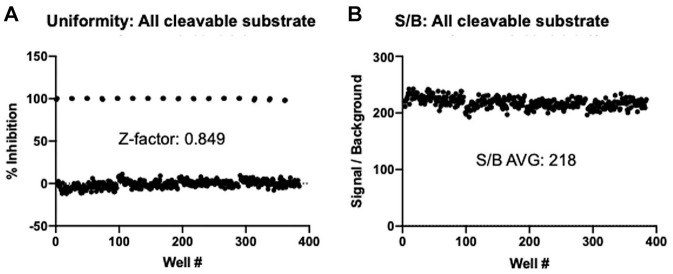
Robustness of SAMDI-MS assay for NSP14/NSP10 ExoN activity using all cleavable substrate. Full-plate uniformity data using optimized conditions and 6 µL reaction volumes for SAMDI-MS highlight the (**A**) robustness measured by the Z factor and (**B**) assay window measured the S/B ratio. Low controls were prequenched with 100 mM EDTA (final).

The robustness of the SAMDI-MS assay was assessed in 6 µL volumes in 384-plate format using the optimized conditions. Columns 1 and 2 of each plate included 100% inhibition controls by prequenching with 100 mM EDTA (final). The presence of positive and negative controls permitted calculation of a Z factor (a measure of robustness), which considers the mean and standard deviation for positive and negative controls. The Z factor using the all cleavable substrate is 0.849 (**Fig. 4A**), with an average S/B ratio of 218 (**Fig. 4B**). Similarly, the SAMDI-MS is robust using the “Exo control” with a Z factor of 0.823 and an average S/B ratio of 107 (**Suppl. Fig. S4**). This significant S/B ratio measured by SAMDI-MS is attributed to the distinct mass of the nuclease product(s) and the internal standard. These data converge on kinetically relevant conditions to identify small-molecule inhibitors of NSP14/NSP10 ExoN activity using SAMDI-MS.

### HTS of NSP14/NSP10 ExoN Activity

**Figure 5.**
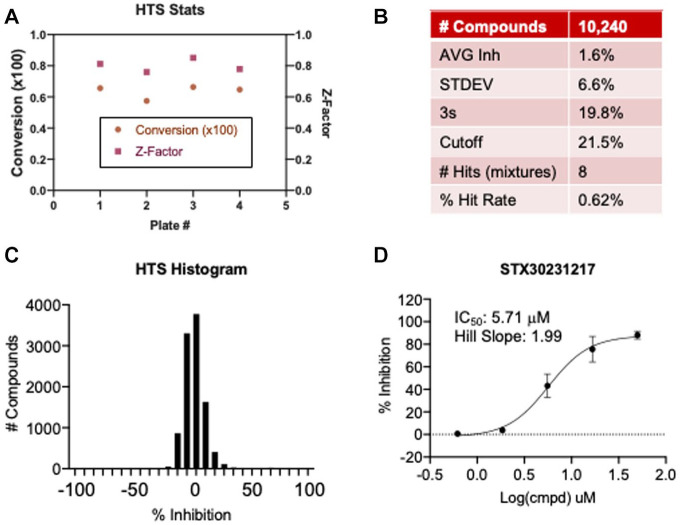
HTS data. (**A**) Total percent conversion of Exo all cleavable substrate to products in DMSO control wells and the Z factor across each plate screened. (**B**) Statistics for the HTS. (**C**) Histogram showing the percent inhibition for all compounds analyzed in the screen. (**D**) The inhibition of compound STX30231217 identified in the HTS is evaluated in a dose–response format against NSP14/NSP10 ExoN activity by SAMDI-MS.

To demonstrate the screening capability of the SAMDI-MS NSP14 ExoN assay, 10,240 compounds from a diverse library were screened for inhibitors of NSP14. This diverse set was synthesized in 2020 and features an average purity >95%. Each compound is dissolved in DMSO, and the library was pooled with eight compounds per well prior to analysis, where each compound was tested at a final concentration of 12.5 µM and a final DMSO concentration of 1%. The robustness of the assay performed well across all four plates, with consistent activity and an average Z factor of 0.800 (**Fig. 5A**). The threshold for determining a hit was calculated by summing the average inhibition across the screen (1.6%) with three times the standard deviation (6.6%) of the average inhibition across the screen. The robustness of the assay therefore allows a hit cutoff of 21.5% inhibition, resulting in eight wells, or a 0.62% hit rate (**Fig. 5B,C**). To identify the hits, the eight compounds present in each pooled hit were analyzed individually in a dose–response format (50, 16.6, 5.55, 1.85, 0.62 µM) in duplicate. At least one compound was confirmed as a hit in seven out of eight pooled compound wells (87.5% validation rate), with one compound, STX30231217, exhibiting an IC_50_ of 5.7 µM (**Fig. 5D**). Given the propensity for RNA intercalation, the hits were tested in an RNA intercalation assay using Thiazole Orange and compared with a mitoxantrone control (**Suppl. Fig. S5A,B**). The data confirm that it does not inhibit through RNA intercalation (**Suppl. Fig. S5C**). Moreover, the compound exhibits minimal activity against RNaseT1, suggesting selective behavior toward NSP14/NSP10 ExoN activity (**Suppl. Fig. S5D**). These data highlight the identification of a novel NSP14/NSP10 inhibitor and support the representation of the SAMDI-MS assay as a powerful and attractive platform for HTS to identify NSP14/NSP10 ExoN inhibitors.

## Discussion

This study reports, to the best of our knowledge, the first label-free and high-throughput assay to measure NSP14/NSP10 ExoN activity and opens avenues for accelerated and improved drug discovery efforts to find small-molecule inhibitors against COVID-19. The SAMDI-MS technology has recently proven to be a powerful approach for measuring SARS-CoV-2 3CLpro activity,^32^ including simultaneously reporting on SARS-CoV-2 3CLpro and rhinovirus HRV3C protease activities.^33^ In addition to the SARS-CoV-2 target, the SAMDI-MS technology has been reported to characterize dozens of biochemical activities, including posttranslational modifying enzymes,^24,34–37^ RNA-modifying proteins,^30^ and arginase,^29^ highlighting its flexibility and broad drug discovery solutions. SAMDI-MS offers several solutions over other MS approaches, including traditional MALDI. A major challenge of MALDI is that without a sample cleanup step, buffer components such as salts and detergents induce ion suppression, thereby impacting data quality. This is critical for enzymes such as nucleases that require MgCl_2_ for activity. Conversely, the SAMDI-MS technology is amenable to any buffer system due to the specific immobilization of the analytes to the self-assembled monolayer (**Fig. 1C,D**). This solution allows a true optimized assay condition according to the target, rather than focusing on optimized conditions for the instrument, which may not be shared with the target. Importantly, SAMDI-MS maintains the high-throughput capability of MALDI and detects singly charged, nonfragmented molecules for simplified data interpretation, including small molecules, lipids, peptides, proteins, and oligonucleotides.

The SAMDI-MS assay offers a label-free alternative toward measuring nuclease activities, including radioactivity^7,8,17^ and fluorescence assays.^18^ Eliminating the need for radionuclides offers a solution for handling the hazardous material, along with waste and safety concerns. Without a fluorescent reporter, the SAMDI-MS assay eliminates false positives that result from optical interference of library compounds.^32^ An added benefit of SAMDI-MS is the ability to distinguish multiple products of nuclease activities, which can shed light on the mechanism. This capability also serves to provide quality control in every well to ensure the integrity of the RNA, as RNases present in enzyme preps or in laboratories can impact data and go undetected in label-dependent approaches. Finally, the SAMDI-MS assay is flexible and can be applied to other RNA and DNA nucleases, including exo- and endonucleases, opening avenues for broad drug discovery in the fields of DNA damage repair along with antivirals, and other pathologies. The advantages of the SAMDI-MS assay contribute to the significant Z factors (>0.80) of this assay and the superior S/B (>100) that make it attractive for small-molecule screening.

Another novel aspect of this study is that it represents the first high-throughput assay suitable for initiating small-molecule drug discovery efforts focused on NSP14 ExoN activity. The data provide the first insight into the kinetic parameters, such as the *K*_M_ of the utilized substrate. Indeed, even the most recent studies rely on lower-throughput approaches such as gel electrophoresis with radiolabeled substrates,^3^ supporting the novelty of this approach. The high-throughput capacity enabled the screening of 10,240 compounds and identification of potential hits that exhibit activity (IC_50_ < 10 µM) and selectivity (>10-fold) (**Fig. 5, Suppl. Fig. S5**) over other RNA nucleases, further highlighting the power of the SAMDI-MS technology. An attractive option for determining the selectivity of compounds is the ability to analyze several assays simultaneously, an approach recently used to evaluate the potency and selectivity of inhibitors of the SARS-CoV-2 3CLpro enzyme.^33^ Future work will also aim to evaluate the antiviral effect of the newly identified NSP14 ExoN inhibitors in cells infected with SARS-CoV-2. It is possible that selective NSP14 ExoN inhibitors do not directly inhibit virus replication in cell culture, but instead act in synergy with other classes of SARS-CoV-2 antivirals. Importantly, the Food and Drug Administration-approved nucleoside analog remdesivir is more efficacious against viruses lacking the NSP14 ExoN function, therefore providing a strong rationale for the combination of these two mechanisms of action.^38^ Another goal will be to continue to explore a larger chemical space to identify novel chemical matter for this challenging target, along with opportunities to multiplex the N7-MTase activity of NSP14. In conclusion, the SAMDI-MS technology represents the first label-free and high-throughput assay for screening potential inhibitors of NSP14 ExoN activity. Moreover, it provides significant benefits for sensitivity, selectivity, and robustness required for drug discovery. Lastly, the flexibility of the approach allows SAMDI-MS to report on many biochemical and binding activities,^39^ accelerating drug discovery across challenging targets and disease areas.

## Supplemental Material

sj-pdf-1-jbx-10.1177_24725552211008854 – Supplemental material for Label-Free Screening of SARS-CoV-2 NSP14 Exonuclease Activity Using SAMDI Mass Spectrometry Click here for additional data file. Supplemental material, sj-pdf-1-jbx-10.1177_24725552211008854 for Label-Free Screening of SARS-CoV-2 NSP14 Exonuclease Activity Using SAMDI Mass Spectrometry by Michael D. Scholle, Cheng Liu, Jerome Deval and Zachary A. Gurard-Levin in SLAS Discovery

## References

[1] Gorbalenya A. E. Krupovic M. Mushegian A.; et al. The New Scope of Virus Taxonomy: Partitioning the Virosphere into 15 Hierarchical Ranks. Nat. Microbiol. 2020, 5, 668–674. 32341570 10.1038/s41564-020-0709-x PMC7186216

[2] Fan K. Ma L. Han X.; et al. The Substrate Specificity of SARS Coronavirus 3C-Like Proteinase. Biochem. Biophys. Res. Commun. 2005, 329, 934–940. 15752746 10.1016/j.bbrc.2005.02.061 PMC7092912

[3] Ogando N. S. Zevenhoven-Dobbe J. C. van der Meer Y.; et al. The Enzymatic Activity of the nsp14 Exoribonuclease Is Critical for Replication of MERS-CoV and SARS-CoV-2. J. Virol. 2020, 94, e01246-20. 10.1128/JVI.01246-20 PMC7654266 32938769

[4] Denison M. R. Graham R. L. Donaldson E. F.; et al. Coronaviruses: An RNA Proofreading Machine Regulates Replication Fidelity and Diversity. RNA Biol. 2011, 8, 270–279. 21593585 10.4161/rna.8.2.15013 PMC3127101

[5] Becares M. Pascual-Iglesias A. Nogales A.; et al. Mutagenesis of Coronavirus nsp14 Reveals Its Potential Role in Modulation of the Innate Immune Response. J. Virol. 2016, 90, 5399–5414. 27009949 10.1128/JVI.03259-15 PMC4934755

[6] Minskaia E. Hertzig T. Gorbalenya A. E.; et al. Discovery of an RNA Virus 3′→5′ Exoribonuclease That Is Critically Involved in Coronavirus RNA Synthesis. Proc. Natl. Acad. Sci. U.S.A. 2006, 103, 5108–5113. 16549795 10.1073/pnas.0508200103 PMC1458802

[7] Ma Y. Wu L. Shaw N.; et al. Structural Basis and Functional Analysis of the SARS Coronavirus nsp14-nsp10 Complex. Proc. Natl. Acad. Sci. U.S.A. 2015, 112, 9436–9441. 26159422 10.1073/pnas.1508686112 PMC4522806

[8] Bouvet M. Imbert I. Subissi L.; et al. RNA 3′-End Mismatch Excision by the Severe Acute Respiratory Syndrome Coronavirus Nonstructural Protein nsp10/nsp14 Exoribonuclease Complex. Proc. Natl. Acad. Sci. U.S.A. 2012, 109, 9372–9377. 22635272 10.1073/pnas.1201130109 PMC3386072

[9] Ferron F. Subissi L. Silveira De Morais A. T.; et al. Structural and Molecular Basis of Mismatch Correction and Ribavirin Excision from Coronavirus RNA. Proc. Natl. Acad. Sci. U.S.A. 2018, 115, E162–E171. 29279395 10.1073/pnas.1718806115 PMC5777078

[10] Al-Tawfiq J. A. Memish Z. A. Update on Therapeutic Options for Middle East Respiratory Syndrome Coronavirus (MERS-CoV). Expert Rev. Anti Infect. Ther. 2017, 15, 269–275. 27937060 10.1080/14787210.2017.1271712 PMC7103731

[11] Stockman L. J. Bellamy R. Garner P. SARS: Systematic Review of Treatment Effects. PLoS Med. 2006, 3, e343. 16968120 10.1371/journal.pmed.0030343 PMC1564166

[12] Eckerle L. D. Becker M. M. Halpin R. A.; et al. Infidelity of SARS-CoV Nsp14-Exonuclease Mutant Virus Replication Is Revealed by Complete Genome Sequencing. PLoS Pathog. 2010, 6, e1000896. 20463816 10.1371/journal.ppat.1000896 PMC2865531

[13] Eckerle L. D. Lu X. Sperry S. M.; et al. High Fidelity of Murine Hepatitis Virus Replication Is Decreased in nsp14 Exoribonuclease Mutants. J. Virol. 2007, 81, 12135–12144. 17804504 10.1128/JVI.01296-07 PMC2169014

[14] Smith E. C. Blanc H. Surdel M. C.; et al. Coronaviruses Lacking Exoribonuclease Activity Are Susceptible to Lethal Mutagenesis: Evidence for Proofreading and Potential Therapeutics. PLoS Pathog. 2013, 9, e1003565. 23966862 10.1371/journal.ppat.1003565 PMC3744431

[15] Case J. B. Li Y. Elliott R.; et al. Murine Hepatitis Virus nsp14 Exoribonuclease Activity Is Required for Resistance to Innate Immunity. J. Virol. 2018, 92, e01531-17. 10.1128/JVI.01531-17 PMC5730787 29046453

[16] Narayanan N. Nair D. T. Ritonavir May Inhibit Exoribonuclease Activity of nsp14 from the SARS-CoV-2 Virus and Potentiate the Activity of Chain Terminating Drugs. Int. J. Biol. Macromol. 2020, 168, 272–278. 33309661 10.1016/j.ijbiomac.2020.12.038 PMC7724963

[17] Chen P. Jiang M. Hu T.; et al. Biochemical Characterization of Exoribonuclease Encoded by SARS Coronavirus. J. Biochem. Mol. Biol. 2007, 40, 649–655. 17927896 10.5483/bmbrep.2007.40.5.649

[18] Sheppard E. C. Rogers S. Harmer N. J.; et al. A Universal Fluorescence-Based Toolkit for Real-Time Quantification of DNA and RNA Nuclease Activity. Sci. Rep. 2019, 9, 8853. 31222049 10.1038/s41598-019-45356-z PMC6586798

[19] Gurard-Levin Z. A. Kilian K. A. Kim J.; et al. Peptide Arrays Identify Isoform-Selective Substrates for Profiling Endogenous Lysine Deacetylase Activity. ACS Chem. Biol. 2010, 5, 863–73. 10.1021/cb100088g PMC2941244 20849068

[20] Kuo H. Y. DeLuca T. A. Miller W. M.; et al. Profiling Deacetylase Activities in Cell Lysates with Peptide Arrays and SAMDI Mass Spectrometry. Anal. Chem. 2013, 85, 10635–10642. 24088168 10.1021/ac402614x PMC3912874

[21] Szymczak L. C. Sykora D. J. Mrksich M. Using Peptide Arrays to Profile Phosphatase Activity in Cell Lysates. Chemistry 2020, 26, 165–170. 31691395 10.1002/chem.201904364

[22] Gurard-Levin Z. A. Mrksich M. Combining Self-Assembled Monolayers and Mass Spectrometry for Applications in Biochips. Annu. Rev. Anal. Chem. (Palo Alto Calif.) 2008, 1, 767–800. 20636097 10.1146/annurev.anchem.1.031207.112903

[23] Mrksich M. Mass Spectrometry of Self-Assembled Monolayers: A New Tool for Molecular Surface Science. ACS Nano 2008, 2, 7–18. 19206542 10.1021/nn7004156 PMC2600870

[24] Patel K. Sherrill J. Mrksich M.; et al. Discovery of SIRT3 Inhibitors Using SAMDI Mass Spectrometry. J. Biomol. Screen. 2015, 20, 842–848. 26024947 10.1177/1087057115588512

[25] Kim J. Mrksich M. Profiling the Selectivity of DNA Ligases in an Array Format with Mass Spectrometry. Nucleic Acids Res. 2010, 38, e2. 19854942 10.1093/nar/gkp827 PMC2800213

[26] Anderson L. L. Berns E. J. Bugga P.; et al. Measuring Drug Metabolism Kinetics and Drug-Drug Interactions Using Self-Assembled Monolayers for Matrix-Assisted Laser Desorption-Ionization Mass Spectrometry. Anal. Chem. 2016, 88, 8604–8609. 27467208 10.1021/acs.analchem.6b01750

[27] Anderson S. E. Fahey N. S. Park J.; et al. A High-Throughput SAMDI-Mass Spectrometry Assay for Isocitrate Dehydrogenase 1. Analyst 2020, 145, 3899–3908. 32297889 10.1039/d0an00174k PMC7440924

[28] Helal K. Y. Alamgir A. Berns E. J.; et al. Traceless Immobilization of Analytes for High-Throughput Experiments with SAMDI Mass Spectrometry. J. Am. Chem. Soc. 2018, 140, 8060–8063. 29901996 10.1021/jacs.8b02918 PMC6578359

[29] Scholle M. D. Gurard-Levin Z. A. Development of a Novel Label-Free and High-Throughput Arginase-1 Assay Using Self-Assembled Monolayer Desorption Ionization Mass Spectrometry. SLAS Discov. 2021. DOI: 10.1177/24725552211000677. 33754845

[30] Buker S. M. Gurard-Levin Z. A. Wheeler B. D.; et al. A Mass Spectrometric Assay of METTL3/METTL14 Methyltransferase Activity. SLAS Discov. 2020, 25, 361–371. 31585521 10.1177/2472555219878408

[31] Zhang J. H. Chung T. D. Oldenburg K. R. A Simple Statistical Parameter for Use in Evaluation and Validation of High Throughput Screening Assays. J. Biomol. Screen. 1999, 4, 67–73. 10838414 10.1177/108705719900400206

[32] Gurard-Levin Z. A. Liu C. Jekle A.; et al. Evaluation of SARS-CoV-2 3C-Like Protease Inhibitors Using Self-Assembled Monolayer Desorption Ionization Mass Spectrometry. Antiviral Res. 2020, 182, 104924. 32896566 10.1016/j.antiviral.2020.104924 PMC7834858

[33] Liu C. Boland S. Scholle M. D.; et al. Dual Inhibition of SARS-CoV-2 and Human Rhinovirus with Protease Inhibitors in Clinical Development. Antiviral Res. 2021, 187, 105020. 33515606 10.1016/j.antiviral.2021.105020 PMC7839511

[34] O’Kane P. T. Dudley Q. M. McMillan A. K.; et al. High-Throughput Mapping of CoA Metabolites by SAMDI-MS to Optimize the Cell-Free Biosynthesis of HMG-CoA. Sci. Adv. 2019, 5, eaaw9180. 10.1126/sciadv.aaw9180 PMC6551189 31183410

[35] Swalm B. M. Knutson S. K. Warholic N. M.; et al. Reaction Coupling between Wild-Type and Disease-Associated Mutant EZH2. ACS Chem. Biol. 2014, 9, 2459–2464. 25154026 10.1021/cb500548b

[36] Szymczak L. C. Huang C. F. Berns E. J.; et al. Combining SAMDI Mass Spectrometry and Peptide Arrays to Profile Phosphatase Activities. Methods Enzymol. 2018, 607, 389–403. 30149867 10.1016/bs.mie.2018.04.021 PMC6457119

[37] Wigle T. J. Swinger K. K. Campbell J. E.; et al. A High-Throughput Mass Spectrometry Assay Coupled with Redox Activity Testing Reduces Artifacts and False Positives in Lysine Demethylase Screening. J. Biomol. Screen. 2015, 20, 810–820. 25755264 10.1177/1087057115575689

[38] Agostini M. L. Andres E. L. Sims A. C.; et al. Coronavirus Susceptibility to the Antiviral Remdesivir (GS-5734) Is Mediated by the Viral Polymerase and the Proofreading Exoribonuclease. mBio 2018, 9, e00221-18. 10.1128/mBio.00221-18 PMC5844999 29511076

[39] VanderPorten E. C. Scholle M. D. Sherrill J.; et al. Identification of Small-Molecule Noncovalent Binders Utilizing SAMDI Technology. SLAS Discov. 2017, 22, 1211–1217. 28581894 10.1177/2472555217712761

