# Supplementary material for: Label-Free Screening of SARS-CoV-2 NSP14 Exonuclease Activity Using SAMDI Mass Spectrometry
Source: SLAS Discov. 2021 Apr 17;26(6):766–74. doi: 10.1177/24725552211008854 (PMC8053483; doi:10.1177/24725552211008854)

SUPPLEMENTARY INFORMATION

Label-free screening of SARS-CoV-2 NSP14 exonuclease activity using SAMDI mass spectrometry.

Michael D Scholle[1], Cheng Liu[2], Jerome Deval[2], Zachary A Gurard-Levin[1]#

[1]SAMDI Tech, Inc., Chicago, IL

[2]Aligos Therapeutics, Inc., South San Francisco, CA

# Address correspondence to: zgurardlevin@samditech.com

Keywords:

COVID-19, coronavirus, NSP14, nuclease, mass spectrometry, label-free

Supplementary Figure S1. SAMDI mass spectrometry (MS) assay of NSP14 ExoN activity. The % conversion to product is measured in a time course by SAMDI-MS for (A) dsRNA Exo substrate, (B) ssRNA Exo substrate, (C) dsRNA LS11 substrate, (D) ssRNA LS11 substrate, (E) dsRNA LS12 substrate, and (F) ssRNA LS12 substrate. All measurements from triplicate data.

Supplementary Figure S2. NSP14/NSP10 substrate specificity and reaction mechanism. (A) The amount of each product is measured by SAMDI-MS. (B) Representative SAMDI-MS spectra of NSP14/NSP10 activity before (*top*) and after (*bottom*) the reaction. (C) Sequences corresponding to the observed product peaks in (B). NSP14/NSP10 activity measured in a time course against the Exo control substrate using (D) WT enzyme and (E) NSP14 D273A catalytic mutant. All measurements from triplicate data.

Supplementary Figure S3. Kinetic parameters determined by SAMDI-MS. The amount of total product in nM over a range of substrate concentrations is measured using SAMDI-MS for (A) the Exo control and (B) Exo all cleavable substrates used to determine reaction rates and KM. The linear portion of NSP14/NSP10 activity is measured by SAMDI-MS over a range of enzyme concentrations in a time course using 100 nM (C) Exo control substrate and (D) Exo all cleavable substrates.

Supplementary Figure S4. Robustness of SAMDI-MS assay for NSP14/NSP10 ExoN activity using the Exo control substrate. Full plate uniformity data using optimized conditions and 6 μL reaction volumes for SAMDI-MS highlights (A) robustness measured by Z-factor and (B) assay window measured by signal to background ratio. Low controls were pre-quenched with 100 mM EDTA (final).

Supplementary Figure S5. High-throughput screening data. (A) RNA intercalation of STX30231217 is measured by a Thiazole Orange assay. (B) Control for RNA intercalation determined using mitoxantrone. (C) RNaseT1 activity measured by SAMDI-MS over a range of RNaseT1 concentrations shows linear activity. (D) STX30231217 is tested in a dose response against RNaseT1 activity.

Supplementary Figure 1

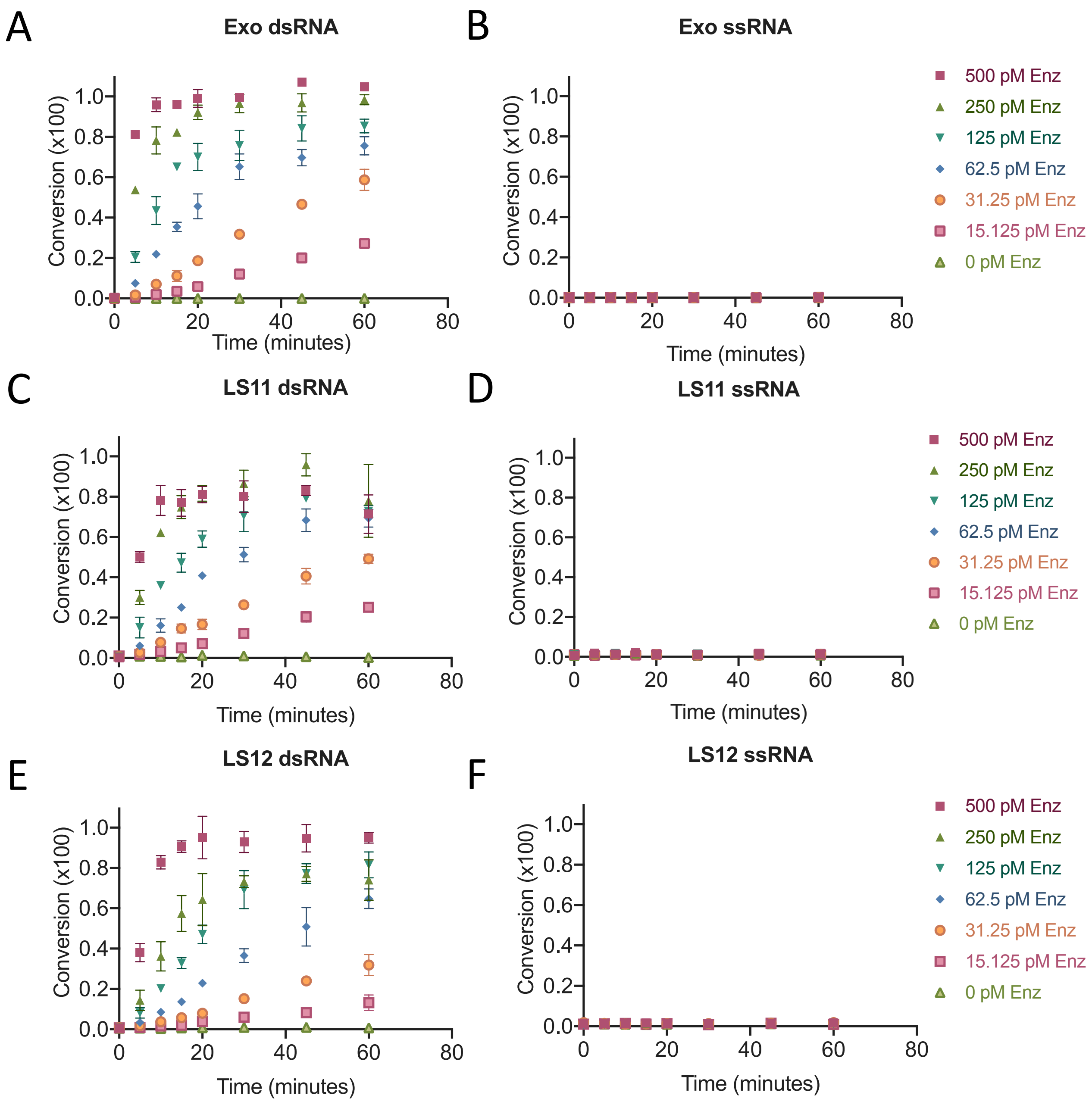

Supplementary Figure 2

A

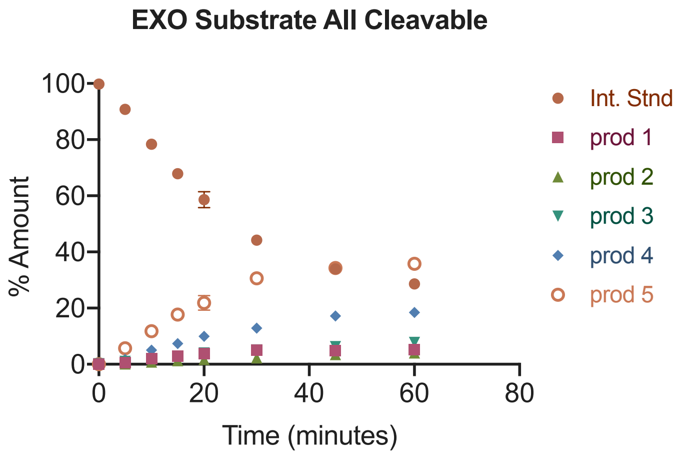

B

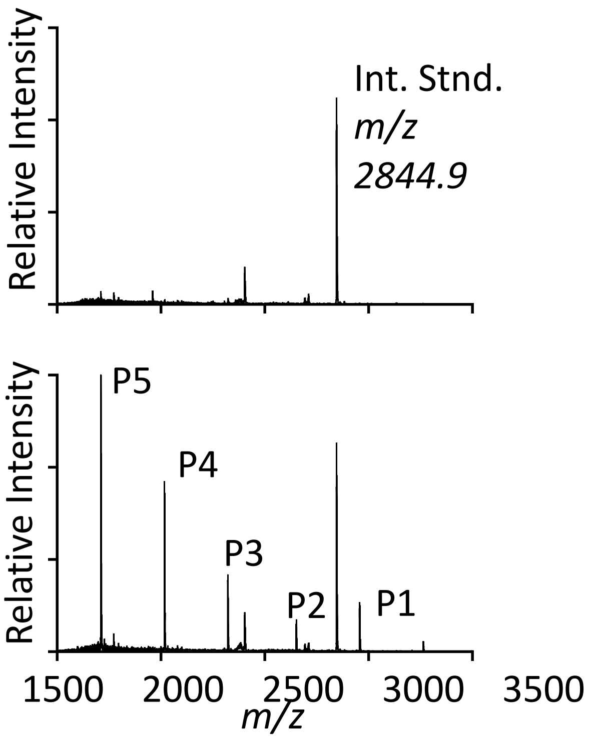

C

Name of RNA	Product Sequence
P1	Biotin-5'-AACAUCA-3'
P2	Biotin-5'-AACAUC-3'
P3	Biotin-5'-AACAU-3'
P4	Biotin-5'-AACA-3'
P5	Biotin-5'-AAC-3'

D

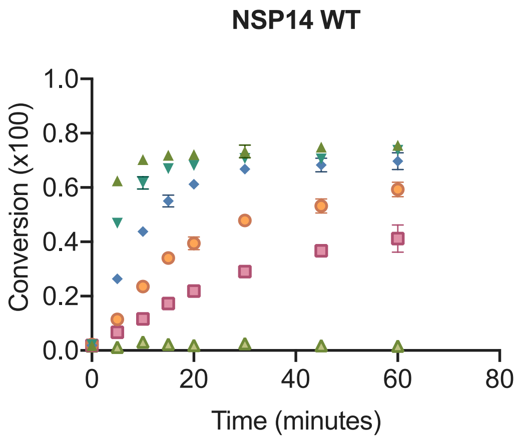

E

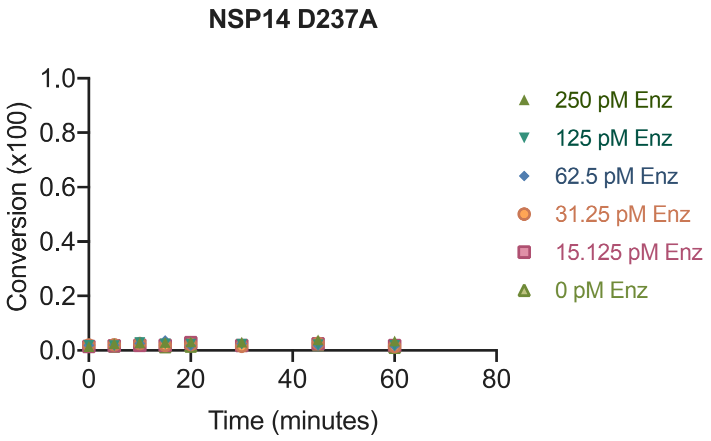

Supplementary Figure 3

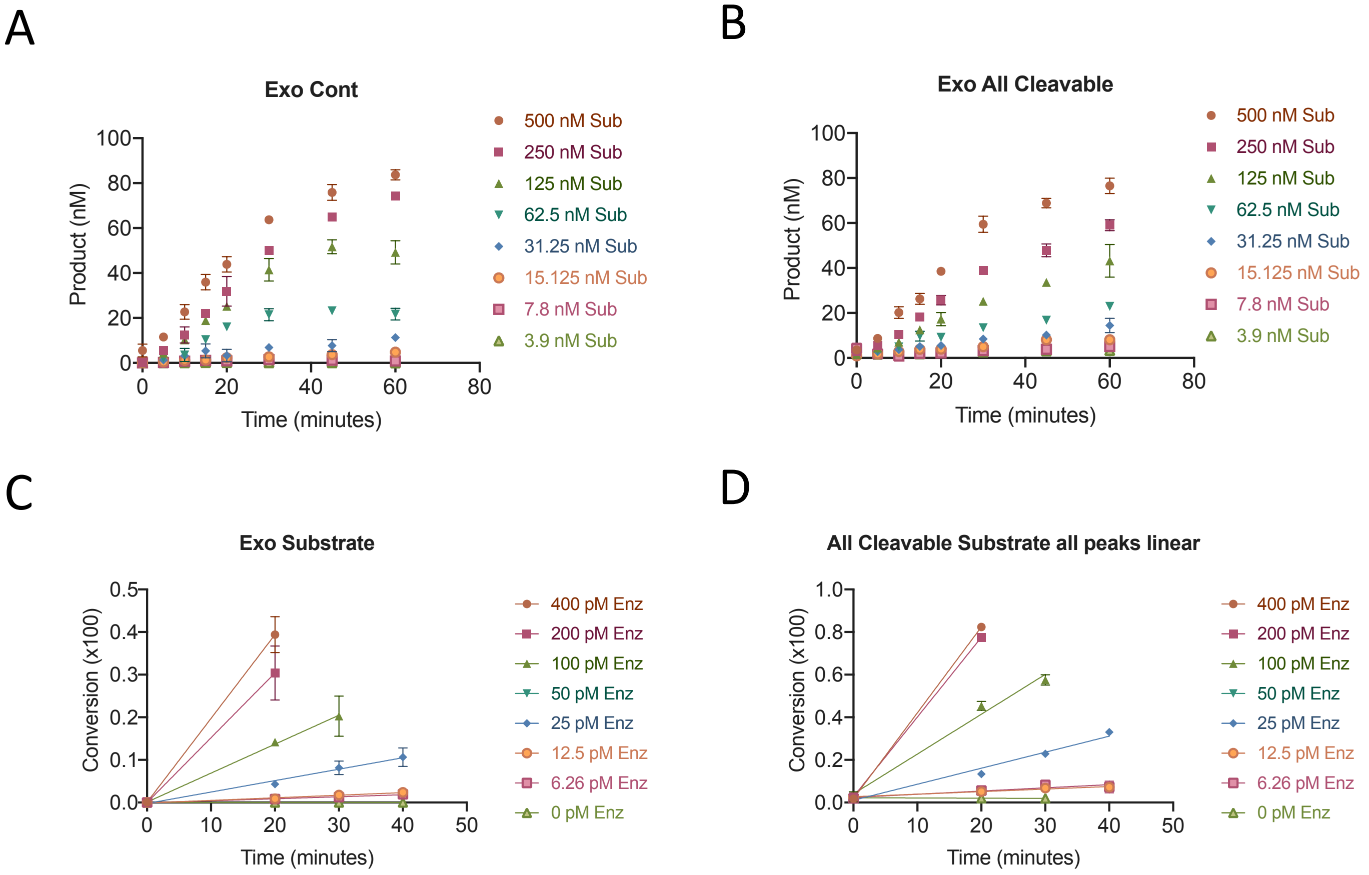

Supplementary Figure 4

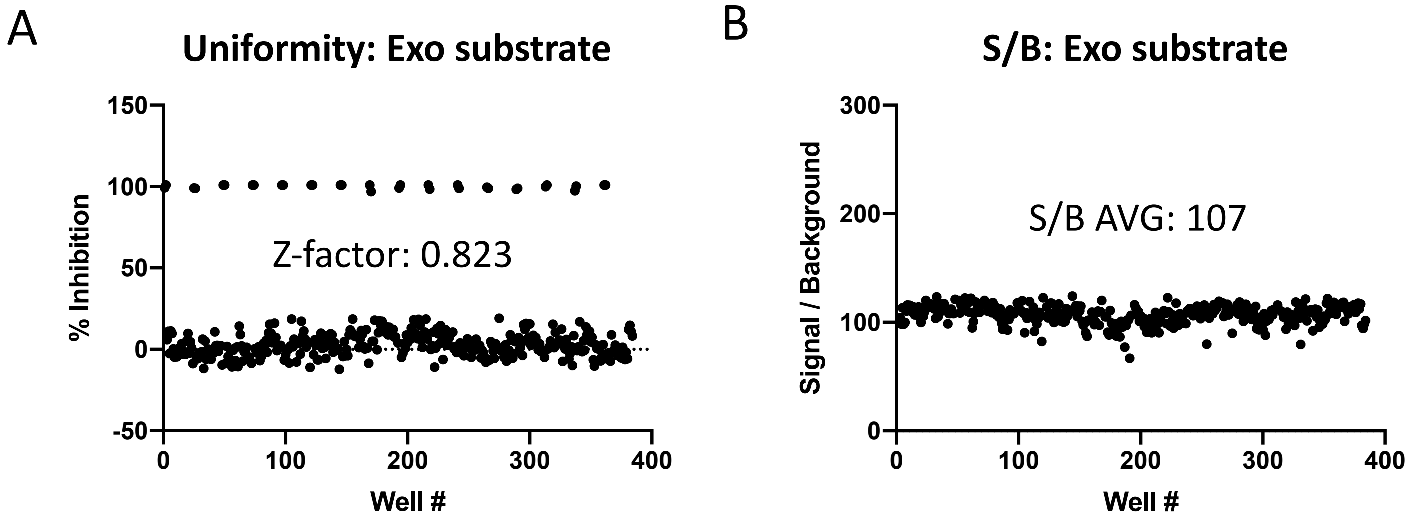

Supplementary Figure 5

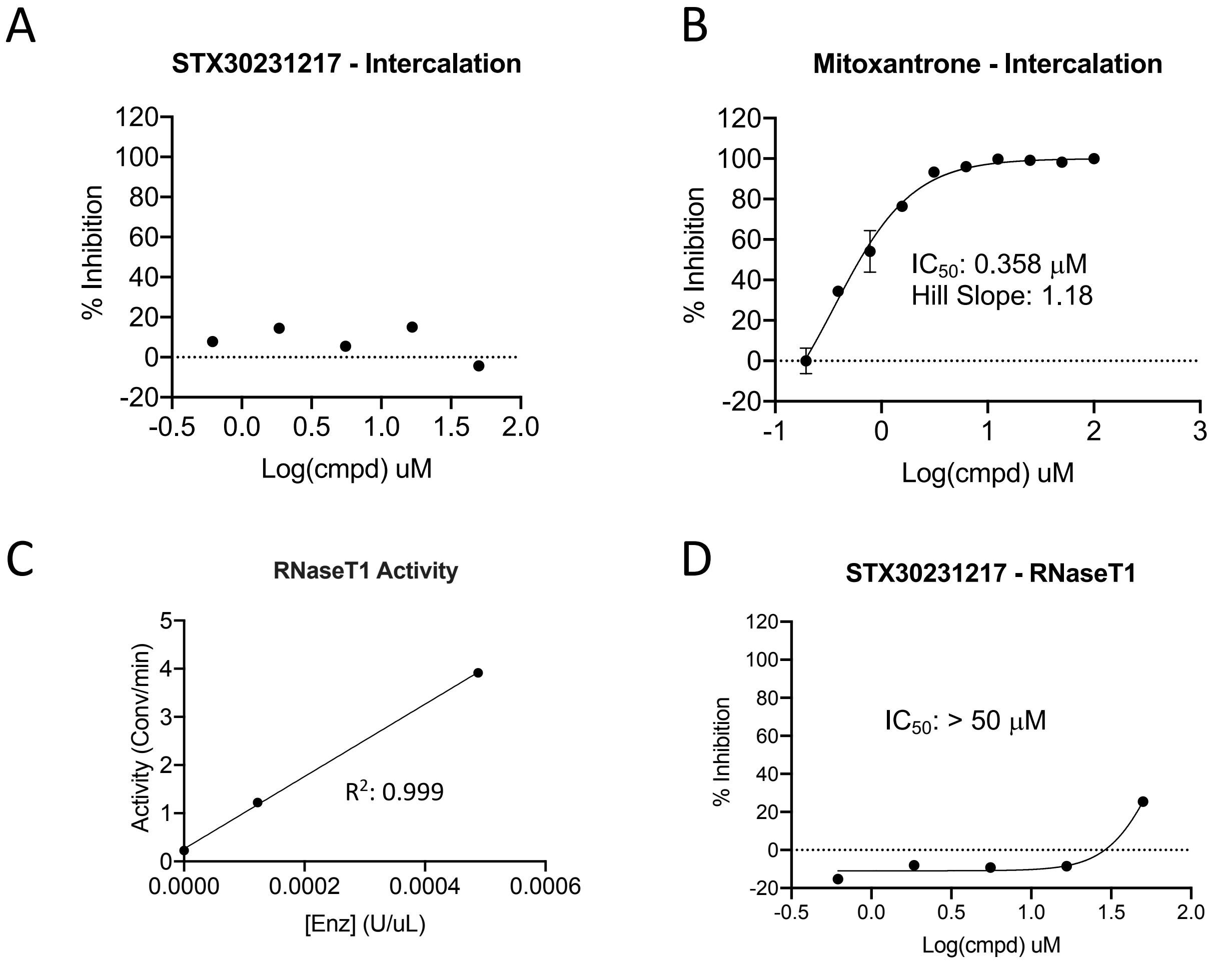

Supplement: sj-pdf-1-jbx-10.1177_24725552211008854 – Supplemental material for Label-Free Screening of SARS-CoV-2 NSP14 Exonuclease Activity Using SAMDI Mass Spectrometry [file sj-pdf-1-jbx-10.1177_24725552211008854.pdf]
